# Membrane filter removal in FTIR spectra through dictionary learning for exploring explainable environmental microplastic analysis

**DOI:** 10.1038/s41598-024-70407-5

**Published:** 2024-08-31

**Authors:** Suphachok Buaruk, Pattara Somnuake, Sarun Gulyanon, Somrudee Deepaisarn, Seksan Laitrakun, Pakorn Opaprakasit

**Affiliations:** 1 Sirindhorn International Institute of Technology, Thammasat University, Pathum Thani, 12120 Thailand; 2 College of Interdisciplinary Studies, Thammasat University, Pathum Thani, 12120 Thailand; 3 Thammasat University Research Unit in Sustainable Electrochemical Intelligent, Thammasat University, Pathum Thani, 12120 Thailand

**Keywords:** Infrared spectroscopy, Computational chemistry, Environmental monitoring

## Abstract

Microplastic analysis is a crucial step for locating the environmental contamination sources and controlling plastic contamination. A popular tool like Fourier transform infrared (FTIR) spectroscopy is capable of identifying plastic types and can be carried out through a variety of containers. Unfortunately, sample collection from water sources like rivers usually involves filtration so the measurements inevitably include the membrane filter that also has its own FTIR characteristic bands. Furthermore, when plastic particles are small, the membrane filter’s spectrum may overwhelm the desired plastics’ spectrum. In this study, we proposed a novel preprocessing method based on the dictionary learning technique for decomposing the variations within the acquired FTIR spectra and capturing the membrane filter’s characteristic bands for the effective removal of these unwanted signals. We break down the plastic analysis task into two subtasks — membrane filter removal and plastic classification — to increase the explainability of the method. In the experiments, our method demonstrates a 1.5-fold improvement compared with baseline, and yields comparable results compared to other state-of-the-art methods such as UNet when applied to noisy spectra with low signal-to-noise ratio (SNR), but offers explainability, a crucial quality that is missing in other state-of-the-art methods. The limitations of the method are studied by testing against generated spectra with different levels of noise, with SNR ranging from 0 to – 30dB, as well as samples collected from the lab. The components/atoms learned from the dictionary learning technique are also scrutinized to describe the explainability and demonstrate the effectiveness of our proposed method in practical applications.

## Introduction

Plastic contamination in the environment poses a multifaceted threat to ecosystems. Plastics break down into microplastics (MPs), particles smaller than 5 mm^1^, increasingly found in natural water and soil, posing risks to the food chain. Studying this issue involves collecting samples, sample treatments with contamination control, and MP identification^2^. Identifying MPs is crucial for understanding their impact and scope on the environment and human health, allowing researchers to assess distribution, sources, and potential pathways through ecosystems. This information is vital for formulating effective mitigation strategies, policies, and technologies to reduce MP pollution and its adverse effects on aquatic life, food chains, and overall environmental quality^3^.

One widely utilized technique for MP identification is Fourier transform infrared (FTIR) spectroscopy^4^, leveraging the highly specific infrared (IR) spectra to reveal distinct band patterns of specific plastics. This method enables the verification of the synthetic plastic origin of potential MPs and provides information on the physico-chemical weathering of plastic particles by detecting the intensity of oxidation^5^. FTIR spectroscopy has found applications in various studies, e.g., MP analysis of sediment samples from the North Sea utilizing micro-FTIR spectroscopy^5^, MP monitoring in a wastewater treatment plant using reflectance micro-FTIR imaging^6^, and MP detection in commercial mussels^7^. The use of FTIR spectroscopy in the analysis of MPs in water has also been emphasized, as it represents a novel mainstream method offering both qualitative and quantitative analysis of MPs^8–11^.

In MP analysis of water samples, membrane filters play a crucial role in the process of capturing and concentrating MP particles present in the water through the filtration process^12^. In spectral acquisition, the membrane filter is usually examined under FTIR to identify and quantify MP particles that are adhered to its surface. The issue is that these membrane filters also show FTIR characteristic bands of their own, so the acquired spectra are the combination of both the MPs of interest and the membrane filters. The identification process is simple when particle sizes are in millimeters. However, for particles in micrometers, the spectra of MP are overwhelmed by the membrane filter’s spectrum. This complicates the identification process and sometimes renders the process challenging even for the experts.

Researchers commonly approach MP identification through supervised learning by employing classification tasks due to the distinct FTIR spectral patterns exhibited. Machine learning (ML) methods such as K-Nearest Neighbors (KNN), Support Vector Machine (SVM)^13^, and Random Forest (RF)^14^ are popular choices for this purpose, as seen in previous studies^15–17^. Recent trends favor deep learning techniques, particularly convolutional neural networks (CNNs)^18–20^ and recurrent neural networks (RNNs)^21,22^ and transformers^23^, for automated feature extraction from raw spectra and producing classification results superior to the aforementioned ML methods. Nevertheless, the limitation of the classification approach lies in its black-box nature, especially for noisy spectra, where producing only the prediction of the types of MPs is insufficient. Understanding results in terms of physical processes or chemical interactions of MPs is essential for gaining meaningful insights into reducing MP pollution. This can be achieved by recovering the MP spectra. However, the main obstacle is the interfering spectrum, such as that from membrane filters. Breaking the task into smaller subtasks like reconstruction and classification of the MP spectra enhances the ability to comprehend these insights^24^. In our MP analysis of water samples, spectral classification of noisy data involves a preprocessing step to remove the membrane filter’s spectrum, the main complicating factor for spectral reconstruction, and a classification step recognizing spectral signatures to distinguish different plastic types.

Hence, the crucial preprocessing step to remove the membrane filter’s spectrum ensures accurate analysis of MPs in water samples. Various methods for membrane filter removal include treating the spectrum as noise and employing denoising techniques such as autoencoders (AE)^25^ in these works^26,27^ and the UNet architecture^28^ like these studies^29,30^. Considering the distinct band patterns in FTIR spectra of plastic polymers, which are indicative of various chemical bonds and functional groups, we anticipate the model to learn these patterns. However, AE and UNet methods use dense representation, having only desirable mathematical properties and lacking meaningful representation in the problem domain. An alternative is a sparse representation, which utilizes a small number of non-zero elements or features in a higher-dimensional space for a more interpretable and compact representation. The sparse representation is more suitable as it aligns better with the characteristics of the problem^31^, where there are many distinct band patterns and only a small number of patterns are present. This technique often leads to a smaller set of significant features that may capture distinct patterns meaningful to chemists.

A popular method for finding sparse representation is dictionary learning, which aims to represent data as a sparse linear combination of basis elements or atoms^32^. It learns a set of atoms that captures underlying structures and patterns, enabling efficient and compact representations^33^. Through iterative updates using optimization algorithms like gradient descent or alternating minimization, dictionary learning transforms high-dimensional data into a lower-dimensional representation that emphasizes salient features for tasks like denoising, compression, and classification. It has been applied to many spectral analysis tasks such as low-rank matrix approximation to speed up data acquisition^34^ and introduced a classification method achieving higher accuracy with lower computational cost^35^.

This study investigates the capabilities, limitations, and benefits of sparse representation for membrane filter removal. We employ dictionary learning to introduce a novel approach for membrane filter removal. A data-centric approach is adopted to create a dataset, enabling dictionary learning to effectively extract components resembling functional group information used by chemists. These components serve to identify, remove, and reconstruct the spectra affected by the membrane filter. Our novel dictionary-learning-based method was applied to both measured and synthetic FTIR spectral data, and its performance was assessed by comparing it to state-of-the-art (SOTA) methods, such as AE^25^ and UNet^28^. Our method demonstrated comparable or superior results with explainability. Its capability was evaluated across different levels of signal-to-noise ratio (SNR), showing slower deterioration compared to other methods as SNR decreases. The benefits of our method were explored through the examination of atom profiles or components extracted by dictionary learning, revealing valuable information for chemists and the identification task.

## Results

### FTIR spectra

**Figure 1:**
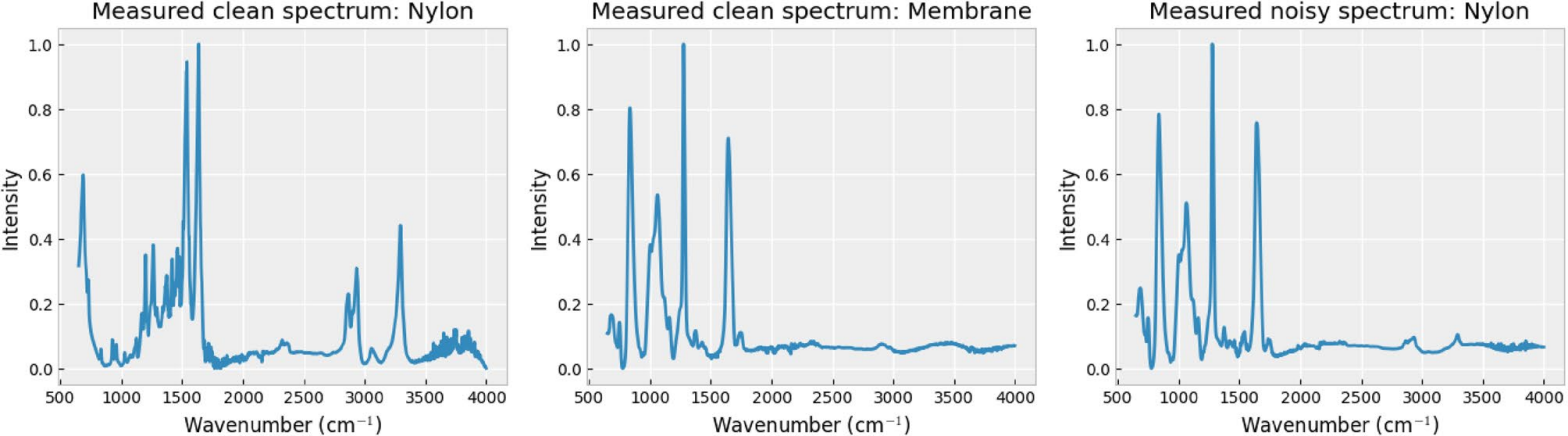
FTIR spectra from the three measured datasets used in this work — measured clean spectra dataset, measured membrane filter spectra dataset, and measured noisy spectra dataset (from left to right).

In this research, we investigate 22 types of plastics, namely Cellulose, high-density polyethylene (HDPE), low-density polyethylene (LDPE), polycarbonate (PC), polyetheretherketone (PEEK), polyoxymethylene (POM), polypropylene (PP), polytetrafluoroethylene (PTFE), polyvinyl chloride (PVC), polyvinyl alcohol (PVA), Acrylic, Nylon, poly(butylene succinate) (PBS), poly(ethylene terephthalate) (PET), polylactide (PLA), polybutylene adipate terephthalate (PBAT), ethylene propylene diene monomer rubber (EPDM), epoxidized natural rubber (ENR), polyethylenimine (PEI), polymethyl methacrylate (PMMA), polyurethane (PU), and polystyrene (PS). The objective of the spectral analysis conducted in this study is to discriminate the chemical composition of neat MP materials from MPs adhered to a membrane filter substrate, to assist in the removal of the membrane filter’s spectrum and the MP identification. To obtain the dataset for supporting a supervised learning approach with input-label sample pairs, the FTIR spectra-gathering process involves three separate datasets under varied conditions, where the intensity of all spectra is normalized to enhance the training efficiency and performance of the methods (Fig. 1):

1. Measured clean spectra dataset: This involves obtaining 10 spectra per MP type using a controlled setup, exclusively measuring the MP material. These spectra serve as a reference for the pure material’s spectral signature.
2. Measured membrane filter spectra dataset: Thirty spectra are collected by measuring only the membrane filter. This dataset is designed to help comprehend the spectral characteristics of the membrane filter substrate.
3. Measured noisy spectra dataset: Sixty spectra for each MP type are acquired by depositing MPs onto membrane filters. This dataset simulates the spectra we expect to obtain from the analysis of water field samples.

### Data synthesis

**Figure 2:**
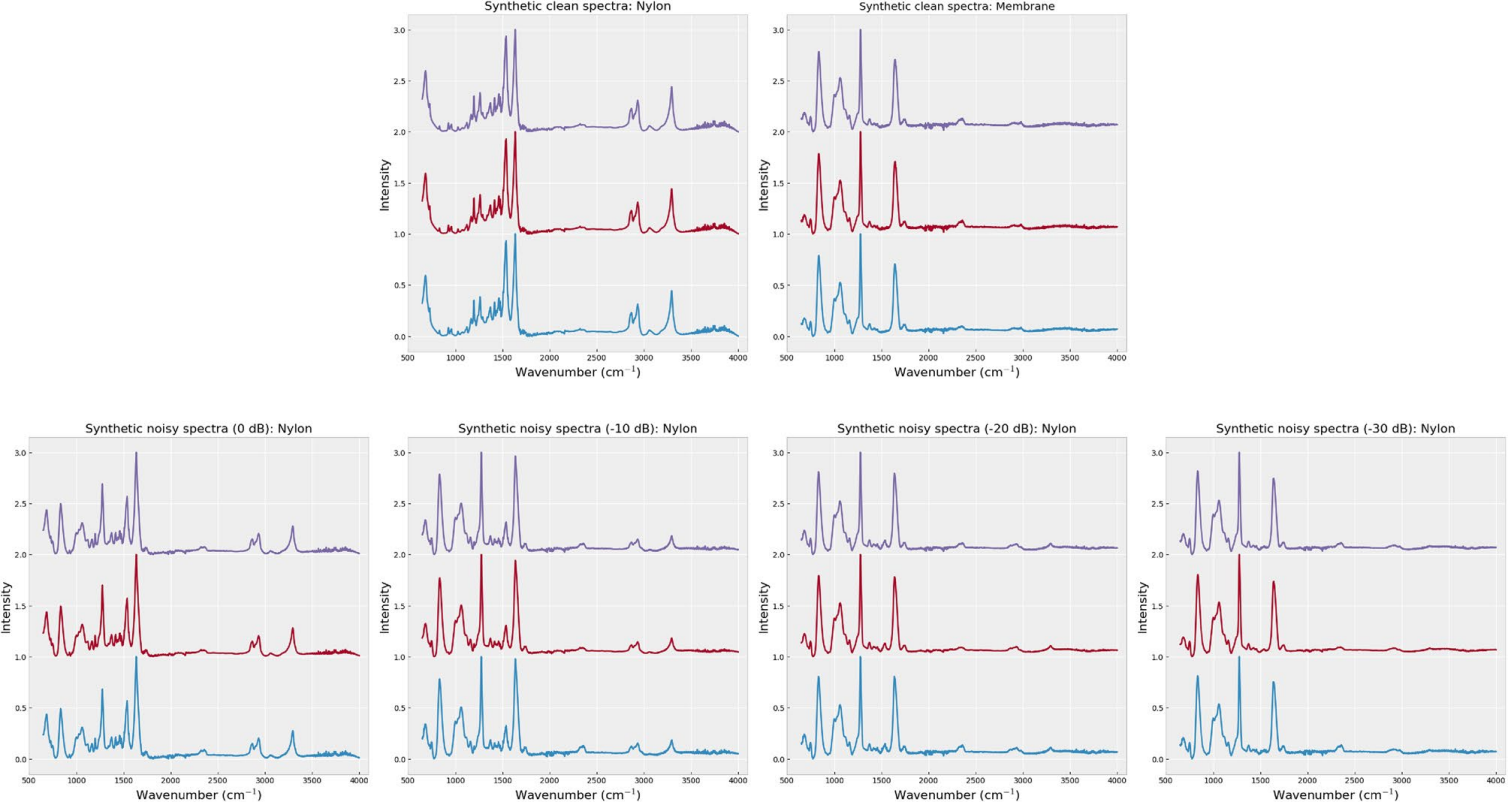
FTIR spectra from the three synthetic datasets used in this work — synthetic clean spectra dataset (top-left), synthetic membrane filter spectra dataset (top-right), and synthetic noisy spectra dataset (bottom). (Bottom, from left to right) FTIR spectra from the synthetic noisy spectra dataset with SNR at 0dB, – 10dB, – 20dB, and – 30dB, respectively.

To examine the capability of our membrane filter removal method, the evaluation is carried out across various SNR levels. Due to the limited number of acquired spectra, obtaining spectra with specific SNR levels can be challenging, so data synthesis is needed. We adopted the data synthesis method similar to that used in this study^36^. The simulation of noisy spectra from MP adhered to membrane filters involves three steps (Fig. 2).

First, we generate a synthetic clean spectra dataset, denoted as ${\textbf{Y}}$. This dataset results from a weighted summation of two randomly normalized spectra of the same MP type from the measured clean spectra dataset. Each synthetic clean spectrum is normalized to ensure that its intensity is between zero and one. These synthetic spectra aim to replicate variations observed in the spectra of pure materials.

Second, we synthesize a dataset of synthetic membrane filter spectra, denoted as ${\textbf{Z}}$. This dataset is created through a weighted summation of two randomly normalized spectra from the measured membrane filter spectra dataset, simulating variations in the membrane filter’s spectra. Each synthetic membrane filter spectrum is also normalized.

Finally, a synthetic noisy spectrum for the *b*-th MP type, denoted as ${\textbf{s}}^{(b)}$, is obtained by combining a synthetic clean spectrum for the *b*-th MP type (${\textbf{y}}^{(b)} \in {\textbf{Y}}$) with a synthetic membrane filter spectrum (${\textbf{z}} \in {\textbf{Z}}$):

1 $$\begin{aligned} {\textbf{s}}^{(b)} = {\textbf{y}}^{(b)} + \beta {\textbf{z}}, \end{aligned}$$

where $\beta$ represents the amplitude scale of the synthetic membrane filter spectrum, ensuring that the SNR (in the unit of dB) of ${\textbf{s}}^{(b)}$ is equal to $-20 \log _{10}(\beta )$. The synthetic clean spectrum ${\textbf{y}}^{(b)}$ serves as the ground truth for the denoising task. All synthetic spectra (${\textbf{s}}^{(b)}$) are normalized by the min-max normalization as well.

It is important to note the division of the measured clean spectra dataset and measured membrane filter spectra dataset into two equal groups. One group is designated for training our model and other ML models, while the other is reserved for model evaluation. This separation ensures the independence of training and test data, originating from distinct sets used in data synthesis.

### MP identification performance

Our dictionary-learning-based method is compared against the SOTA denoising methods, i.e., AE^25^ and UNet^28^. The performances in MP identification are measured by the accuracy of classification results, a percentage of spectra with correctly predicted MP type. Our method and others perform classification by assigning the same material type as the synthetic clean spectrum with the maximum gradient correlation to the denoised spectra.

In all experiments, the learning matrix used in our method consists of 100 synthetic membrane filter spectra from ${\textbf{Z}}$ and 10 synthetic clean spectra per class from ${\textbf{Y}}$. The number of components or atoms is set to 50 and the regularization parameter is set to 1.0. The orthogonal matching pursuit (OMP)^37^ technique was used to learn the dictionary.

The AE uses a multilayer perceptron as the backbone architecture. It consists of two dense layers with 512 and 256 units on the encoder side, with LeakyReLU^38^ as the activation function. Similarly, on the decoder side, there are two dense layers with 256 and 512 units, with LeakyReLU as the activation function. The output layer utilizes the tanh activation function.

The UNet model is modified to use 1D convolutional layers instead of 2D convolutional layers, as in the original work^28^. The UNet model comprises four encoder blocks followed by a middle block and closure with four decoder blocks. The ReLU activation function is used, with the number of filters ranging from 16 to 128 and a kernel size of $5 \times 1$. The output layer is a 1-D CNN layer with a kernel size of $3 \times 1$ and a tanh activation function.

Additionally, we include a baseline method, representing results without any preprocessing, computed by assigning the same material type as the clean spectrum with the maximum gradient correlation to the noisy spectra.

We validated our approach using the measured noisy spectra dataset, representing instances from real-world scenarios. The outcomes of our proposed method, AE, UNet, and the baseline method, are presented in Table 1. For AE and Unet, the synthetic datasets between 0 and – 30dB SNR were used as the training data since these methods require noisy spectra as the input and clean spectra as the ground truth. The findings suggest that while our method performs similarly to other methods in real-world scenarios, the superior explainability inherent in our method enhances the robustness of the results, contributing to its stability across different levels of SNR.

**Table 1:** The accuracy of our method and others on the measured noisy spectra dataset.

Baseline	AE	UNet	Our method
0.4356	0.4333	0.6659	0.6477

To investigate the limitations and robustness of our method, all methods are evaluated on the synthetic noisy spectra dataset at different levels of SNR, i.e., 0dB, – 10dB, – 20dB, and – 30dB. At each level of SNR, the test set consists of 100 synthetic spectra per MP type. The classification results in Table 2 show that our method achieves higher accuracy at low SNR levels (between – 10dB and – 30dB). Although our method is outperformed by AE at high SNR, i.e., at 0dB, it demonstrates more stable behavior. UNet and AE exhibit an undesirable and significant drop of 10% or more in accuracy between – 20dB and – 30dB. While our method may have lower representation power, leading to lower performance at high SNR, it proves to be more robust with consistent accuracy as SNR decreases. Additionally, our method boasts simplicity in terms of training procedure, computational cost, and model size compared to other methods. The training procedure of our method differs from other supervised learning techniques like AE and UNet. Our method relies solely on clean spectra obtained in the laboratory for training, whereas supervised learning methods typically require both training and test sets stemming from the same distribution or a procedure to simulate this, such as data augmentation. This process often requires the collection and analysis of actual water field samples to obtain the ground truth and effectively train models.

**Table 2:** The accuracy of our method and others on the test set at different levels of SNR.

SNR	Baseline	AE	UNet	Our method
0dB	0.9700	0.9877	0.9545	0.9545
– 10dB	0.8355	0.9536	0.9495	0.9545
– 20dB	0.1914	0.9209	0.9377	0.9545
– 30dB	0.0586	0.3164	0.8305	0.9582

### Quality of spectral reconstruction

**Figure 3:**
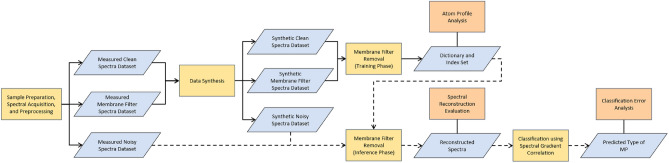
The flowchart illustrates the key steps of our method (yellow boxes) and the analysis of our method, particularly the dictionary learning process related to environmental MP analysis (orange boxes). The solid arrows represent the training phase of dictionary learning, while the dashed arrows indicate the inference phase.

The primary objective may be to detect the presence of MPs, but various aspects of the dictionary learning process and the results from its downstream tasks need to be discussed (Fig. 3). One notable aspect is the ability to reconstruct spectra after membrane filter removal, which can significantly aid chemists in comprehending and endorsing the method’s predictions by increasing its explainability. To measure the quality of the reconstructed spectra, we compute the gradient correlation between the reconstruction of the synthetic noisy spectrum and the corresponding synthetic clean spectrum. Ideally, the reconstructed spectrum should mirror the synthetic clean spectrum, since the synthetic noisy spectrum is a composite of the membrane filter spectrum and the synthetic pure MP spectrum. The gradient correlation value can serve as an indicator of the similarity between the two spectra.

The average of the maximum gradient correlations over the test set is reported in Table 3. The results show that our method yields high-quality reconstructed spectra, exhibiting a higher gradient correlation compared to other methods. Moreover, as SNR decreases, the gap between our method and others widens. Between 0db and – 30dB, the gradient correlation of baseline, AE, and UNet drops around 64%, 43%, and 34% respectively, while our method drops only by 24%. At – 30dB SNR, our method outperforms the others by 18% or more in gradient correlation. This suggests that the results from our method deteriorate at a lower rate compared to other methods, indicating the robustness of our method in the denoising task.

**Table 3:** The gradient correlation of our method and others on the test set at different levels of SNR.

SNR	Baseline	AE	UNet	Proposed method
0dB	0.6995	0.8054	0.9122	0.9856
– 10dB	0.3204	0.8950	0.7037	0.9616
– 20dB	0.1247	0.8850	0.7026	0.9135
– 30dB	0.0576	0.3707	0.5649	0.7451

**Figure 4:**
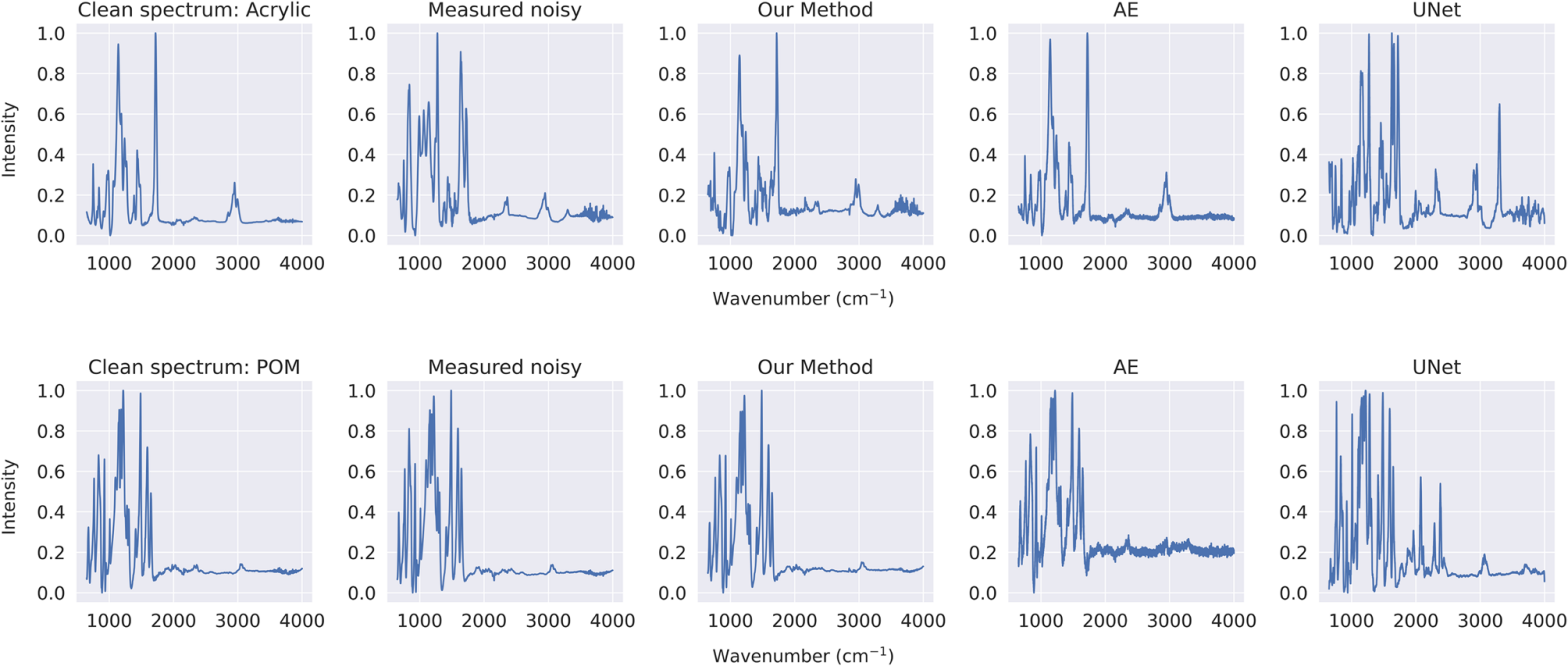
(From left to right) the ground truth/measured clean spectrum, measured noisy spectrum, and the reconstructed spectra using dictionary learning, AE, and UNet, respectively, of measured noisy Acrylic spectrum (top) and POM spectrum (bottom).

Figure 4 visually compares the spectral reconstruction of measured noisy spectra using our dictionary learning, AE, and UNet. The ground truth or the measured clean spectra are given as the reference of what reconstructed spectra should be. Our method demonstrates superior reconstruction quality since the results closely resemble the ground truth. On the other hand, the reconstructed spectra from AE may capture large peaks accurately but they introduce signal fluctuations, which are undesirable for chemists and make it challenging to recognize small peaks. While, UNet fails to eliminate some membrane filter peaks, causing the remaining peaks to mix with the peaks of the MPs and complicating the differentiation of peaks and the MP identification for chemists. Hence, both the qualitative findings in Fig. 4 and the quantitative results in Table 3 indicate that the level of explainability inherent in our method correlates positively with its robustness.

### Atom profile analysis

**Figure 5:**
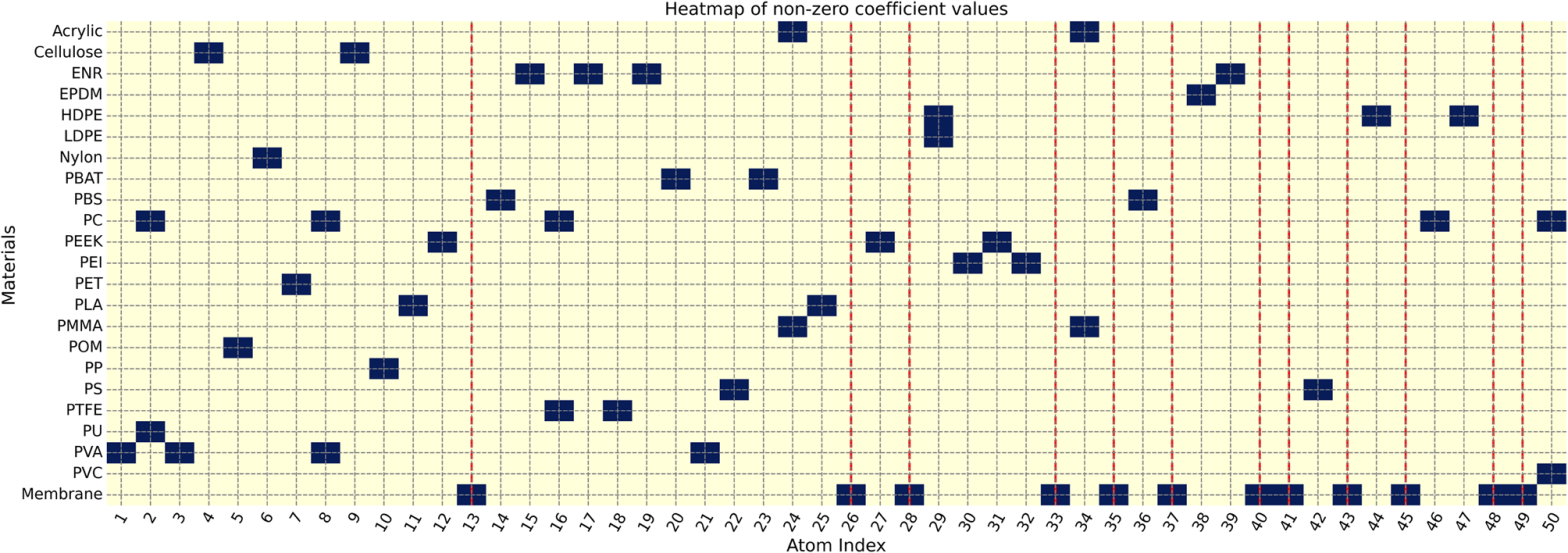
The heatmap of non-zero coefficients (non-zeros in dark blue and zeros in yellow) of 50 atoms across all 22 MPs and the membrane filter spectra from the learning matrix ${\textbf{X}}$. The red dashed lines indicate the atom indices of the membrane filter, which do not overlap with the atom indices of the other MPs.

The key component of our methodology is the atoms or components learned by the dictionary learning, where a spectrum can be expressed by a weighted sum of these atoms. The portion of a spectrum captured by each atom is referred to as the atom profile. These atoms aim to capture distinctive patterns from spectra in the learning matrix ${\textbf{X}}$, which consists of the synthetic clean spectra and the synthetic membrane filter spectra. These learned atoms play a crucial role in reconstructing the spectra and illustrating the spectrum reconstruction ability. Dictionary learning employs sparsity-inducing penalties, which encourage a sparse or minimal set of non-zero coefficients of these atoms. The sparse coefficient matrix offers a concise representation of the spectra in the learning matrix ${\textbf{X}}$. The coefficient values signify the relative importance of atoms within the learned dictionary for each spectrum. Figure 5 illustrates the non-zero coefficients of all spectra in the learning matrix for each material type, demonstrating that most material types respond to a different set of atoms, especially for the membrane filter. This highlights the desirable property of our method that aids in characterizing the membrane filter removal and reconstruction processes.

**Figure 6:**
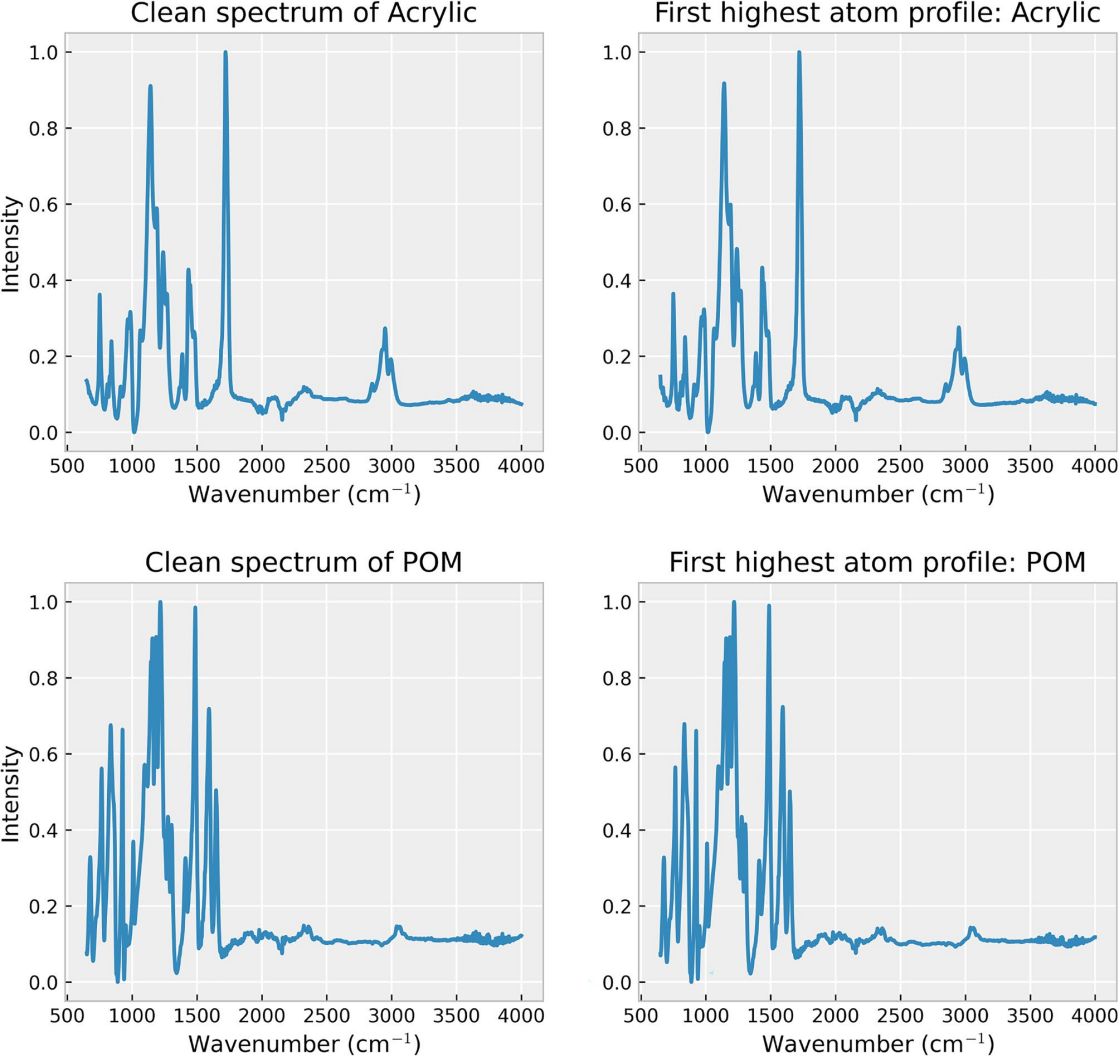
Comparison of the original spectrum (left) with the profile of the highest coefficient atom (right) of the Acrylic (top) and POM (bottom).

**Figure 7:**
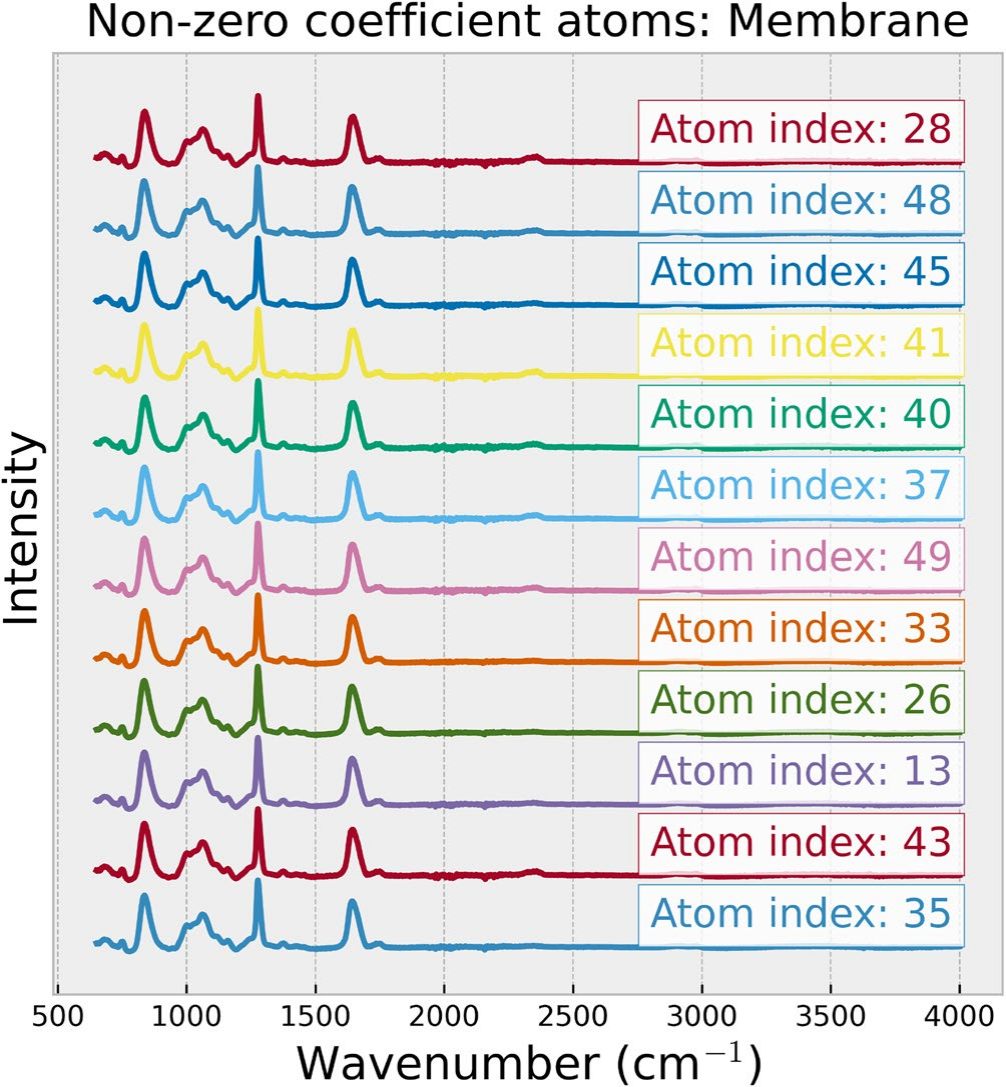
The non-zero coefficient atoms are presented according to the atom profiles of the membrane filter, sorted by atom’s coefficients in descending order from top to bottom.

**Figure 8:**
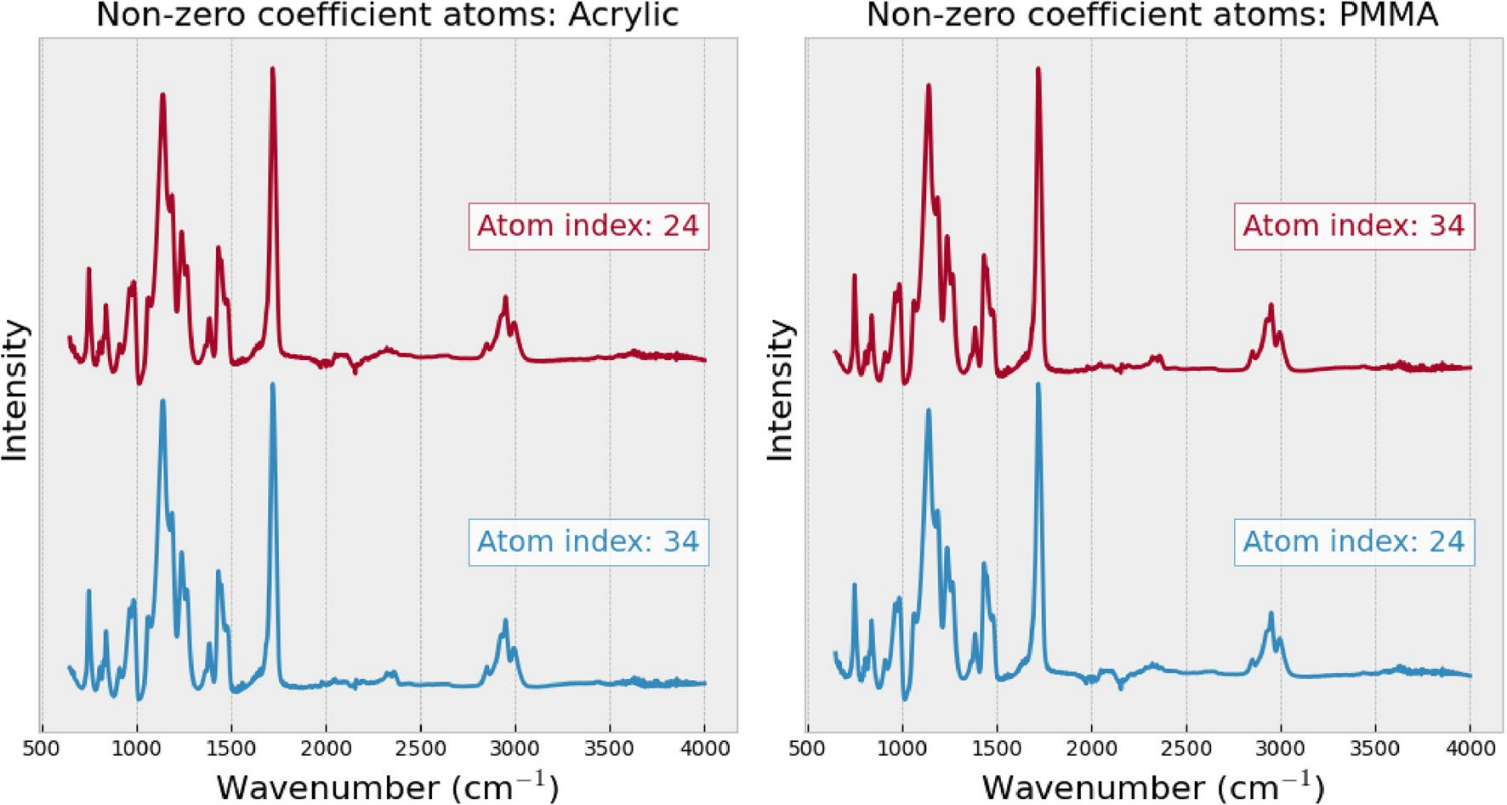
The non-zero coefficient atoms are presented according to the atom profiles of the Acrylic (left) and PMMA (right) spectra, sorted by atoms’ coefficients in descending order from top to bottom.

**Figure 9:**
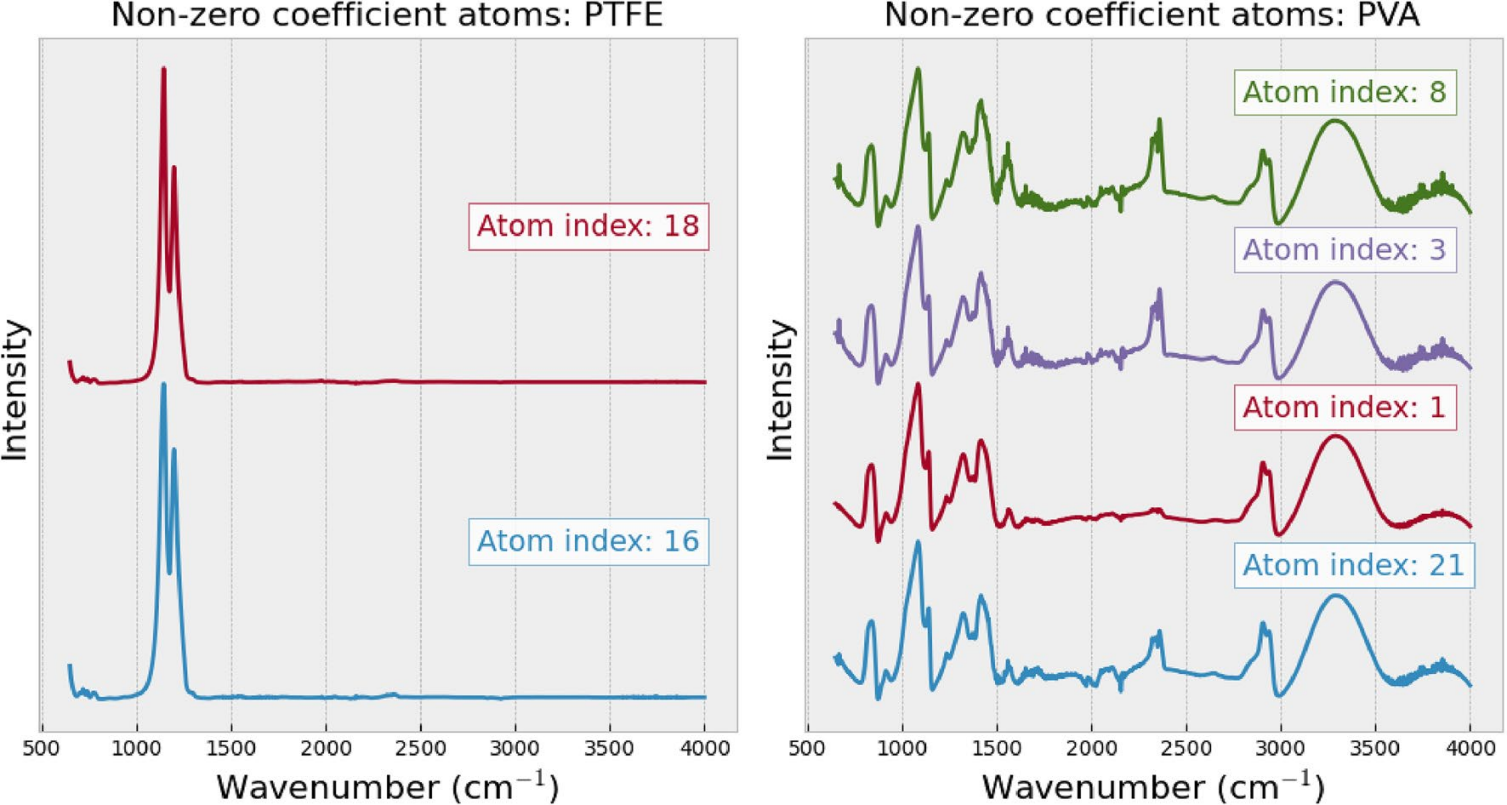
The non-zero coefficient atoms are presented according to the atom profiles of the PTFE (left) and PVA (right) spectra, sorted by atoms’ coefficients in descending order from top to bottom.

**Figure 10:**
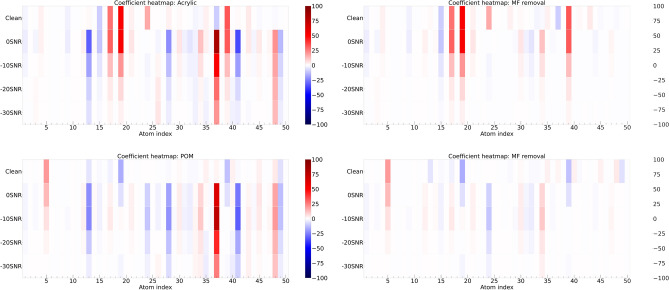
Heatmap of coefficient values in noisy and reconstructed spectrum with SNR at 0dB, -10dB, -20dB, and -30dB, respectively, of the Acrylic (top) and POM (bottom) spectra calculated by dictionary learning technique (left) and after removing coefficients of the membrane filter following our method (right).

Analyzing the atom profiles highlights the highly desirable property in the practical application of our method, which is the explainability of the spectrum reconstruction process. Two key observations in atom profiles shed light on how our method achieves membrane filter spectrum removal and spectrum reconstruction:

1. The profile of the atom with the highest coefficient captures the unique features in MP spectra for all types, as shown in Fig. 6. This dominant atom appears to capture significant information aligned with the distinctive patterns presented in different MP types. This observation suggests the meaningful association between this specific atom and the distinct characteristics of each MP type. It emphasizes the method’s capacity to capture and represent the unique features of each MP type through atoms computed by dictionary learning. Additionally, the profile of the atom associated with the membrane filter resembles the unique pattern of the membrane filter spectrum, as shown in Fig. 7, which is distinguishable from the atom profile of MPs in Figs. 8 and 9. This property enables our method to separate the membrane filter spectrum from the input spectrum.
2. The coefficient values can indicate the presence of MPs and membrane filters in the spectrum. In Fig. 10, as the SNR decreases, the coefficient values of atoms corresponding to MPs (at index 24 for Acrylic and index 5 for POM) decrease. This suggests that when the level of noise overwhelms the MP signal, the coefficients corresponding to the occurrence of MP diminish. At the same time, the coefficient values of atoms corresponding to the membrane filter (e.g., at indices 13, 28, 37, and 41) emerge when the level of noise is high at SNR between 0dB and -30dB (Fig. 10). Reducing the influence of coefficients related to the membrane filter enhances the performance of MP spectrum reconstruction. By excluding these membrane-filter-related coefficients, the remaining information is primarily related to MPs, which facilitates the effective reconstruction of MP spectra.

**Figure 11:**
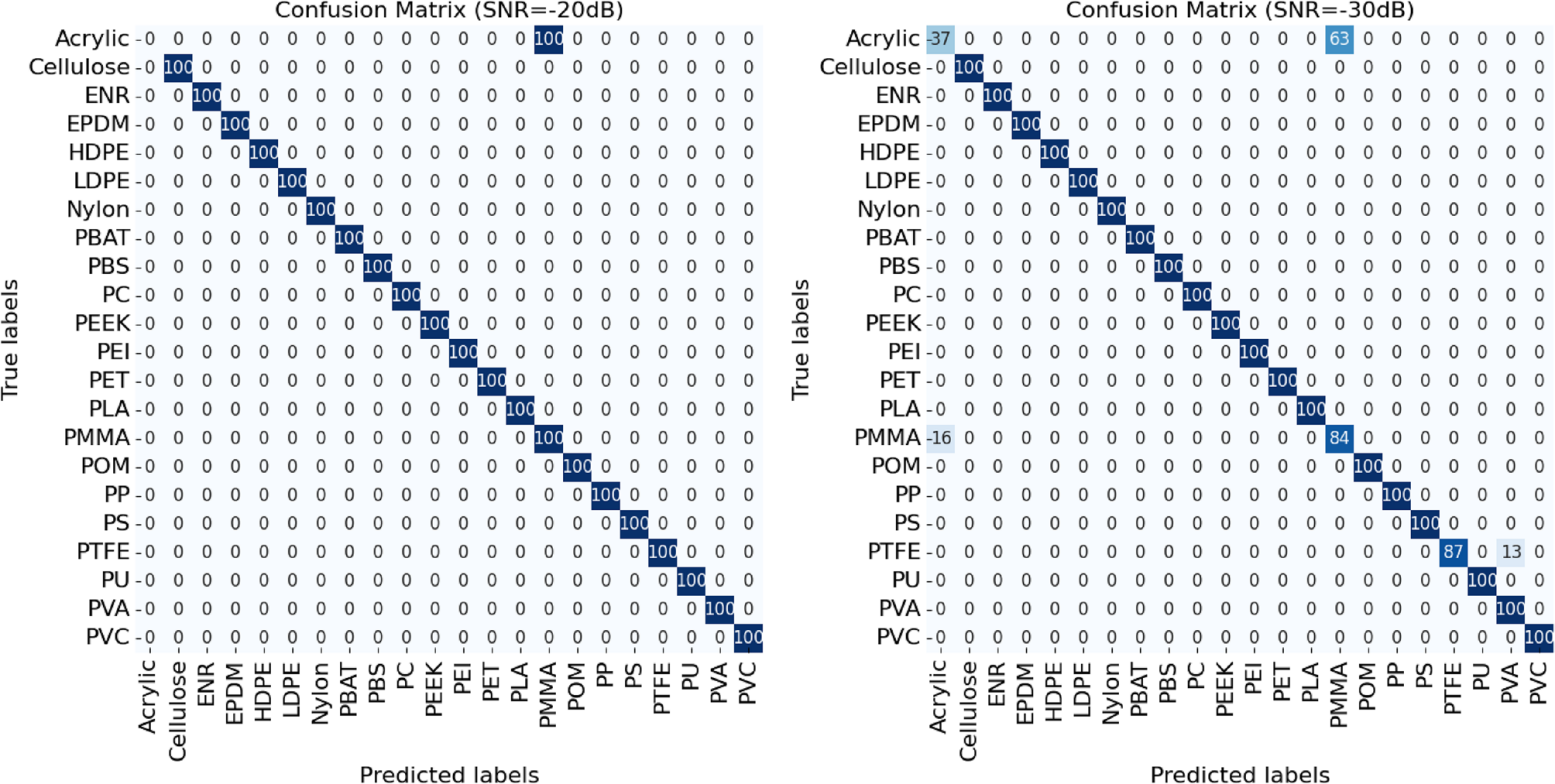
Confusion matrix on the results using our method on the synthetic noisy spectra dataset at SNR of -20dB (left) and -30dB (right).

### Classification error analysis

We examine the limitations of our methodology through an error analysis of the classification performance, focussing on cases where our method failed to accurately identify MPs. This examination begins with the computation of the confusion matrix on the synthetic noisy spectra dataset at SNR of -20dB (Fig. 11 (left)). The result highlights a pair of materials that our method often confuses one with another — Acrylic–PMMA. Addressing this confusion involves exploring of the atom profiles extracted from each type of MP. Figure 8 depicts the atom profiles ranked by their significance of Acrylic and PMMA, revealing noticeable similarities among these atom profiles, as they share the same set of atoms. In fact, the spectra of these MP pairs are also similar to each other, posing a challenging task even for experts attempting to differentiate between them. Apart from these tricky cases, our method accurately classifies 21 out of the 22 types of MPs in the experiment.

We further investigate the atom profiles to explain why our method gives incorrect predictions for the pair of MPs, i.e., Acrylic–PMMA. The potential for confusion during the prediction process is made evident as the reconstruction of the MP pair utilizes the same set of atoms (Fig. 8). It turns out that the atom profiles of both Acrylic and PMMA share the same set of atoms but with different orders of coefficients (atom indexes 24 and 34). This confusion can be further explained in terms of the chemical properties of the polymers involved. PMMA is actually one of the structural variants of Acrylic materials. Therefore, the FTIR characteristics bands of both classes are almost indistinguishable, even for experts.

However, when examining the confusion matrix on the synthetic noisy spectra dataset at SNR of -30dB (Fig. 11 (right)), our method starts misclassifying another pair of materials — PTFE–PVA. This error can be attributed to the overwhelming noise present in the spectra, combined with the fact that PTFE exhibits a low number of peaks (Fig. 9), some of which coincide with peaks in the membrane filter (particularly in the wavenumber range of 1000–1200). As a result, when the peaks of these two materials are merged due to noise, they become indistinguishable in some cases, leading our method to recognize them as peaks of MPs. Consequently, our method selects spectra that bear a higher resemblance to the MF, such as PVA, which possesses a greater number of peaks (Fig. 9). Moreover, our method also starts getting confused between Acrylic–PMMA, unlike before when they were just indistinguishable, resulting in all being categorized as PMMA. While this change may improve accuracy, it suggests that our method has reached its limitation and can no longer effectively differentiate between Acrylic and PMMA or classify PTFE accurately.

## Conclusions

In this study, we introduce a novel approach for membrane filter removal in FTIR spectra based on dictionary learning. Our method is evaluated and compared against two SOTA deep learning models, i.e., autoencoder and UNet, on MP identification and spectrum reconstruction tasks. Evaluation is conducted on both synthetic and experimentally obtained noisy spectra datasets. The results demonstrate that our method achieves comparable classification accuracy to the SOTA method, specifically UNet, and exhibits higher robustness even under conditions of very low SNR. Moreover, by dividing the classification problem into reconstruction and classification steps, our method offers insights into MP spectra that the traditional classification approaches cannot provide. This information is vital for chemists when considering the adoption of AI solutions. Furthermore, our approach has the potential to be applied to other types of filters, beyond the cellulose filter papers used in the experiments, by supplying a new dataset of filter spectra. We anticipate that our proposed method will contribute to making the analysis of MPs from water samples more efficient, accurate, and practicable.

## Methods

**Table 4:** Sample preparation methods of all 22 types of microplastics used in this study.

Polymer types	Sample preparation method
Cellulose, HDPE, LDPE, PC, PEEK, POM, PP, PTFE, PVC, PVA	Polymer (0.05 g, obtained through cryogenic grinding if applicable) was dispersed in IPA (5 ml) and dropped onto a membrane filter.
Acrylic, Nylon, PBS, PET	Polymer (0.05 g, cut from a commercial source or refined with mortar) was dispersed in IPA (5 ml) and dropped onto a membrane filter.
PLA, PBAT, EPDM, ENR, PEI, PMMA	Polymer (0.05 g) was dissolved in CHL (5 ml) and dispersed in water before being dropped onto a membrane filter.
PU	Commercial PU emulsion was dropped onto a membrane filter.
PS	Commercial expanded polystyrene (EPS) was cut and dissolved in CHL, which was subsequently emulsified and dropped onto a membrane filter.

### Sample preparation

The sample preparation process carried out in this study aims to replicate the presence of MPs that may be encountered in water samples. The selected 22 types of plastic samples comprehensively represent groups of common plastics used in everyday life, which become MPs that contaminate the environment. Table 4 describes the procedures for preparing 22 types of plastics, each associated with specific polymer groups. These preparations can be categorized into five approaches. Polymers like Cellulose, HDPE, and LDPE were cryogenically ground and dispersed in isopropyl alcohol (IPA) before being deposited onto a membrane filter. Similarly, Acrylic, Nylon, PBS, and PET followed a similar procedure. PLA, PBAT, EPDM, and others were dissolved in chloroform (CHL) before dispersing in water and being placed on the membrane. Commercial PU emulsion was directly deposited, while PS involved an additional step of dissolving in CHL and emulsifying before depositing on the membrane. The membrane filter employed in this study was supplied by Cytiva, Whatman (Pore size 0.45 micrometer, Diameter 47 mm).

### Spectral acquisition and preprocessing

The process of characterizing the chemical structures of micro- or nano-scaled plastic particles was accomplished through collecting FTIR spectra in an attenuated total reflectance (ATR) mode. The equipment used in this study is the Nicolet iS5 spectrometer (iD7 base, Thermo Scientific, USA). The process includes co-adding 32 scans at a resolution of 2 $\text {cm}^{-1}$ to enhance the quality of the results. This spectroscopic method provides insight into the chemical composition of both neat plastic material and the corresponding plastic particles deposited on a membrane filter substrate^39–42^.

As the spectra are acquired in various experiments, potentially involving changes in calibration setups, there may be variations in the sampled wave number values. To ensure spectral alignment, an interpolation process is applied to guarantee that all spectra are expressed by the same set of wave numbers, specifically 650, 650.5, ..., 3999, 3999.5. Each spectrum is then represented as an $N \times 1$ vector, with *N* set to 6, 700 in our study. Then, a min-max normalization method is applied to each spectrum to normalize the values to a range where the minimum and maximum values are set to zero and one, respectively.

### Membrane filter removal

**Figure 12:**
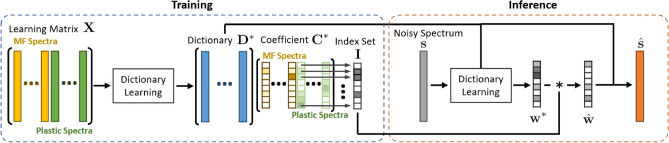
The overview of our membrane filter removal method.

The dictionary learning aims to find a set of basis elements, or atoms, namely a dictionary, that can efficiently represent the spectra dataset. Our method (Fig. 12) adopted a scheme similar to the work proposed in Ref.^43^ that involves learning the dictionary from clean spectra of MPs and membrane filters readily obtainable in the laboratory. Then, this learned dictionary is used to reconstruct the spectra. The process involves two steps. Firstly, we build the atoms that capture the underlying structure of the target spectra, as well as undesired spectra like those from the membrane filter, by applying the dictionary learning to the hand-crafted spectra dataset, referred to as the learning matrix. Then, the atoms corresponding to the membrane filters are rendered irrelevant by setting their coefficients to zeros to eliminate the influence of the membrane filter on the reconstructed spectra.

To ensure the effectiveness of the dictionary, the learning matrix must consist of a diverse range of spectra that expose various patterns of functional groups in FTIR spectra. Moreover, the spectra in the learning matrix must distinctly illustrate these patterns. Therefore, in our study, the learning matrix, ${\textbf{X}}$, includes the clean spectra from all the 22 plastic types available in our dataset, along with the spectra from the membrane filter. It is denoted as follows:

2 $$\begin{aligned} {\textbf{X}}_{N \times M} = \left[ {\textbf{z}}_1, \ldots , {\textbf{z}}_J, {\textbf{y}}_1^{(1)}, \ldots , {\textbf{y}}_L^{(1)}, \ldots , {\textbf{y}}_1^{(22)}, \ldots , {\textbf{y}}_L^{(22)} \right] \end{aligned}$$

where ${\textbf{z}}_j \in {\textbf{Z}}$ is a vector of the membrane filter spectrum and ${\textbf{y}}_l^{(b)} \in {\textbf{Y}}$ is a vector of the clean spectrum of the *b*-th class. The spectrum is represented as an $N \times 1$ vector, where *N* is the number of elements. *J* and *L* are the numbers of membrane filter spectra and clean plastic spectra (per class), respectively. $M = J+22L$ is the total number of spectra in the learning matrix ${\textbf{X}}$. In all experiments, we set *J* to 100 and *L* to 10.

The dictionary learning with the parameter *K*, the number of components or atoms, is applied to the learning matrix ${\textbf{X}}$. This process aims to derive the dictionary ${\textbf{D}}^*$ and the coefficient matrix ${\textbf{C}}^*$ by optimizing the following expression:

3 $$\begin{aligned} \{ {\textbf{D}}^*, {\textbf{C}}^* \} = \arg \min _{{\textbf{D}}, {\textbf{C}}} \left( \frac{1}{2} \Vert {\textbf{X}} - {\textbf{D}} {\textbf{C}} \Vert ^2_2 + \lambda \Vert {\textbf{C}} \Vert _1 \right) \end{aligned}$$

where ${\textbf{D}}^*$ is an $N \times K$ matrix that stores the *K* atoms and each atom has *N* data points. ${\textbf{C}}^*$ is a sparse $K \times M$ matrix of coefficients representing the weights of each atom contributing to each spectrum in the learning matrix ${\textbf{X}}$. $\Vert \cdot \Vert _1$ and $\Vert \cdot \Vert _2$ denote the L1 norm and L2 norm respectively. $\lambda$ is a regularization parameter that balances between the data-fitting term and the sparsity-promoting term. The values in ${\textbf{C}}^*$ will be used to identify which atoms belong to the membrane filters. Since the number of points in a spectrum is large ($N=6,700$), the number of atoms (*K*) that we considered in this study is less than *N*, making the corresponding dictionary ${\textbf{D}}^*$ an undercomplete type. In all experiments, we set *K* to 50 and $\lambda$ to 1.0.

To identify the atoms corresponding to the membrane filter’s spectrum, the coefficients of the membrane filter spectra, denoted as ${\textbf{C}}^*_{MF} = [{\textbf{c}}_1,..., {\textbf{c}}_J]$ are examined. For all non-zero entries in ${\textbf{c}}_1, ..., {\textbf{c}}_J$, the corresponding atoms contribute to the membrane filter’s spectra. Hence, the indices of these non-zero entries in ${\textbf{c}}_1,..., {\textbf{c}}_J$ are stored in the index set ${\textbf{I}}$, expressed as:

4 $$\begin{aligned} {\textbf{I}} = \{ i_1,..., i_A \} \end{aligned}$$

where *A* is the number of atoms meeting these conditions, and $i_a$ is the index of these atoms.

To remove the membrane filter’s spectrum from a new noisy spectrum ${\textbf{s}}$, the following steps are taken. First, we compute the coefficient vector ${\textbf{w}}^*$ of ${\textbf{s}}$ using the learned dictionary ${\textbf{D}}^*$ by the following equation.

5 $$\begin{aligned} {\textbf{w}}^* = \arg \min _{{\textbf{w}}} \left( \frac{1}{2} \Vert {\textbf{s}} - {\textbf{D}}^* {\textbf{w}} \Vert ^2_2 + \lambda \Vert {\textbf{w}} \Vert _1 \right) \end{aligned}$$

Then, to remove the influence of the membrane filter spectrum, we set the coefficient of the atoms corresponding to the membrane filters in the index set ${\textbf{I}}$ to zeros. Given the coefficient vector ${\textbf{w}}^* = [w_1^*,..., w_K^*]^T$, the modified coefficient vector $\hat{{\textbf{w}}}$ after removing the membrane filter is denoted by:

6 $$\begin{aligned} \hat{{\textbf{w}}} = [{\hat{w}}_1,..., {\hat{w}}_K]^T \end{aligned}$$

where

7 $$\begin{aligned} {\hat{w}}_k = {\left\{ \begin{array}{ll} 0 & \text {if } k \in {\textbf{I}} \\ w_k^* & \text {if } k \notin {\textbf{I}} \end{array}\right. }. \end{aligned}$$

Finally, we reconstruct the spectrum $\hat{{\textbf{s}}}$ without the membrane filter’s spectrum using the equation.

8 $$\begin{aligned} \hat{{\textbf{s}}} = {\textbf{D}}^* \hat{{\textbf{w}}}. \end{aligned}$$

### Spectral gradient correlation

The microplastics identification task can then be accomplished through the classification of the reconstructed spectra. A straightforward classification method involves assigning the class with the maximum gradient correlation. The predicted class ${\hat{b}}$ of the reconstructed spectrum ($\hat{{\textbf{s}}}$) is obtained from

9 $$\begin{aligned} {\hat{b}} = \mathop {\mathrm {arg\,max}}\limits _b \{ \rho ^{(b)} \} \end{aligned}$$.

The gradient correlation ($\rho ^{(b)}$) measures the Pearson correlation of spectral gradient between the reconstructed spectrum ($\hat{{\textbf{s}}}$) and the ground-truth clean spectrum of the *b*-th class (${\textbf{y}}^{(b)}$). This is preferred since the spectral gradient can quantitatively describe the spectral shapes^44^. It is defined by the equation:

10 $$\begin{aligned} \rho ^{(b)} = \frac{\sum _{n=1}^{N} (\nabla {\hat{s}}_{n} - \overline{ \nabla {\hat{s}}}) (\nabla {\textbf{y}}^{(b)}_n - \overline{ \nabla {\textbf{y}}^{(b)}_n }) }{ \sqrt{ \Big ( \sum _{n=1}^{N}(\nabla {\hat{s}}_{n} - \overline{ \nabla {\hat{s}}})^2 \Big ) \Big ( \sum _{n=1}^{N}(\nabla {\textbf{y}}^{(b)}_n - \overline{ \nabla {\textbf{y}}^{(b)}_n })^2 \Big ) }}, \end{aligned}$$

where $\nabla {\hat{s}}_{n}$ denotes the gradient of the *n*-th element of the reconstructed spectrum $\hat{{\textbf{s}}}$, $\overline{ \nabla {\hat{s}}}$ denotes the average gradient value of the reconstructed spectrum, $\nabla {\textbf{y}}^{(b)}_n$ denotes the gradient of the *n*-th element of the ground-truth clean spectrum, and $\overline{ \nabla {\textbf{y}}^{(b)}_n }$ denotes the average gradient value of the ground-truth clean spectrum. A higher value of $\rho \in [0, 1]$ indicates a more significant similarity between the spectral shapes of the reconstructed spectrum and the ground-truth clean spectrum.

## Data Availability

The datasets generated and analyzed during the current study are available from Pakorn Opaprakasit (pakorn@siit.tu.ac.th) upon reasonable request.

## References

[1] Pivokonsky, M. *et al.* Occurrence of microplastics in raw and treated drinking water. *Sci. Total Environ.* **643**, 1644–1651. 10.1016/j.scitotenv.2018.08.102 (2018). 30104017 10.1016/j.scitotenv.2018.08.102

[2] Cutroneo, L. *et al.* Microplastics in seawater: Sampling strategies, laboratory methodologies, and identification techniques applied to port environment. *Environ. Sci. Pollut. Res.* **27**, 8938–8952. 10.1007/s11356-020-07783-8 (2020). 10.1007/s11356-020-07783-8 PMC7165152 32026372

[3] Kurniawan, T. A. *et al.* Source, occurrence, distribution, fate, and implications of microplastic pollutants in freshwater on environment: A critical review and way forward. *Chemosphere*[SPACE]10.1016/j.chemosphere.2023.138367 (2023). 36907482 10.1016/j.chemosphere.2023.138367

[4] Xu, J.-L., Thomas, K. V., Luo, Z. & Gowen, A. A. FTIR and Raman imaging for microplastics analysis: State of the art, challenges and prospects. *TrAC, Trends Anal. Chem.* **119**, 115629. 10.1016/j.trac.2019.115629 (2019). 10.1016/j.trac.2019.115629

[5] Löder, M. G. & Gerdts, G. Methodology used for the detection and identification of microplastics-a critical appraisal. *Mar. Anthropogenic Litter*[SPACE]10.1007/978-3-319-16510-3_8 (2015). 10.1007/978-3-319-16510-3_8

[6] Tagg, A. S. *et al.* Microplastic monitoring at different stages in a wastewater treatment plant using reflectance micro-FTIR imaging. *Front. Environ. Sci.* **8**, 145. 10.3389/fenvs.2020.00145 (2020). 10.3389/fenvs.2020.00145

[7] Kumar, B. V., Löschel, L. A., Imhof, H. K., Löder, M. G. & Laforsch, C. Analysis of microplastics of a broad size range in commercially important mussels by combining FTIR and Raman spectroscopy approaches. *Environ. Pollut.* **269**, 116147. 10.1016/j.envpol.2020.116147 (2021). 33280916 10.1016/j.envpol.2020.116147

[8] Renner, G., Sauerbier, P., Schmidt, T. C. & Schram, J. Robust automatic identification of microplastics in environmental samples using FTIR microscopy. *Anal. Chem.* **91**, 9656–9664. 10.1021/acs.analchem.9b01095 (2019). 31287674 10.1021/acs.analchem.9b01095

[9] Fan, C., Huang, Y.-Z., Lin, J.-N. & Li, J. Microplastic constituent identification from admixtures by Fourier-transform infrared (FTIR) spectroscopy: The use of polyethylene terephthalate (PET), polyethylene (PE), polypropylene (PP), polyvinyl chloride (PVC) and nylon (NY) as the model constituents. *Environmental Technology & Innovation* **23**, 101798. 10.1016/j.eti.2021.101798 (2021). 10.1016/j.eti.2021.101798

[10] Gao, Z., Chen, L., Cizdziel, J. & Huang, Y. Research progress on microplastics in wastewater treatment plants: A holistic review. *J. Environ. Manag.* **325**, 116411. 10.1016/j.jenvman.2022.116411 (2023). 10.1016/j.jenvman.2022.116411 36274308

[11] Yang, J. *et al.* Microplastics in different water samples (seawater, freshwater, and wastewater): Removal efficiency of membrane treatment processes. *Water Res.* **232**, 119673. 10.1016/j.watres.2023.119673 (2023). 36764106 10.1016/j.watres.2023.119673

[12] Cai, H. *et al.* Microplastic quantification affected by structure and pore size of filters. *Chemosphere* **257**, 127198. 10.1016/j.chemosphere.2020.127198 (2020). 32512329 10.1016/j.chemosphere.2020.127198

[13] Boser, B. E., Guyon, I. M. & Vapnik, V. N. A training algorithm for optimal margin classifiers. In *Proceedings of the fifth annual workshop on Computational learning theory*, 144–152, 10.1145/130385.130401 (1992).

[14] Breiman, L. Random forests. *Mach. Learn.* **45**, 5–32. 10.1023/A:1010933404324 (2001). 10.1023/A:1010933404324

[15] Chen, X. *et al.* Spectroscopic identification of environmental microplastics. *IEEE Access* **9**, 47615–47620. 10.1109/ACCESS.2021.3063293 (2021). 10.1109/ACCESS.2021.3063293

[16] Jin, N. *et al.* Characterization and identification of microplastics using Raman spectroscopy coupled with multivariate analysis. *Anal. Chim. Acta* **1197**, 339519. 10.1016/j.aca.2022.339519 (2022). 35168726 10.1016/j.aca.2022.339519

[17] Valls-Conesa, J. *et al.* Random forest microplastic classification using spectral subsamples of FT-IR hyperspectral images. *Anal. Methods* **15**, 2226–2233. 10.1039/D3AY00514C (2023). 37114762 10.1039/D3AY00514C

[18] Zhu, Z., Parker, W. & Wong, A. Plasticnet: Deep learning for automatic microplastic recognition via FT-IR spectroscopy. *J. Computat. Vis. Imaging Syst.* **6**, 1–3. 10.15353/jcvis.v6i1.3554 (2020). 10.15353/jcvis.v6i1.3554

[19] Jiang, S. *et al.* Using ATR-FTIR spectra and convolutional neural networks for characterizing mixed plastic waste. *Comput. Chem. Eng.* **155**, 107547. 10.1016/j.compchemeng.2021.107547 (2021). 10.1016/j.compchemeng.2021.107547

[20] Ren, L. *et al.* Identification of microplastics using a convolutional neural network based on micro-Raman spectroscopy. *Talanta* **260**, 124611. 10.1016/j.talanta.2023.124611 (2023). 37163925 10.1016/j.talanta.2023.124611

[21] Tran, H.-T. *et al.* Machine learning approaches for predicting microplastic pollution in peatland areas. *Mar. Pollut. Bull.* **194**, 115417. 10.1016/j.marpolbul.2023.115417 (2023). 37639864 10.1016/j.marpolbul.2023.115417

[22] Neto, J. G., Simon, D. A., Figueiredo, K. & Brandão, A. L. Framework for data-driven polymer characterization from infrared spectra. *Spectrochim. Acta A Mol. Biomol. Spectrosc.* **300**, 122841. 10.1016/j.saa.2023.122841 (2023). 37269658 10.1016/j.saa.2023.122841

[23] Barker, M., Willans, M., Pham, D.-S., Krishna, A. & Hackett, M. Explainable detection of microplastics using transformer neural networks. In *Australasian Joint Conference on Artificial Intelligence* (ed. Barker, M.) 102–115 (Springer, 2022). 10.1007/978-3-031-22695-3_8.

[24] Urrutia, F., Calderon, C. & Barriere, V. Deep natural language feature learning for interpretable prediction. In *Proceedings of the 2023 Conference on Empirical Methods in Natural Language Processing* (eds Bouamor, H. *et al.*) 3736–3763 (Association for Computational Linguistics, 2023). 10.18653/v1/2023.emnlp-main.229.

[25] Kramer, M. A. Nonlinear principal component analysis using autoassociative neural networks. *AIChE J.* **37**, 233–243. 10.1002/aic.690370209 (1991). 10.1002/aic.690370209

[26] Brandt, J., Mattsson, K. & Hassellöv, M. Deep learning for reconstructing low-quality FTIR and Raman spectra-a case study in microplastic analyses. *Anal. Chem.* **93**, 16360–16368. 10.1021/acs.analchem.1c02618 (2021). 34807556 10.1021/acs.analchem.1c02618 PMC8674871

[27] Raulf, A. P. *et al.* Deep representation learning for domain adaptable classification of infrared spectral imaging data. *Bioinformatics* **36**, 287–294. 10.1093/bioinformatics/btz505 (2020). 31225858 10.1093/bioinformatics/btz505

[28] Ronneberger, O., Fischer, P. & Brox, T. U-net: Convolutional networks for biomedical image segmentation. arXiv: 1505.04597 (2015).

[29] Guo, S. *et al.* Deep learning for ‘artefact’ removal in infrared spectroscopy. *Analyst* **145**, 5213–5220. 10.1039/D0AN00917B (2020). 32579623 10.1039/D0AN00917B

[30] Zeng, Y., Liu, Z.-Q., Fan, X.-G. & Wang, X. Modified denoising method of Raman spectra-based deep learning for Raman semi-quantitative analysis and imaging. *Microchem. J.* **191**, 108777. 10.1016/j.microc.2023.108777 (2023). 10.1016/j.microc.2023.108777

[31] Wübbeler, G., Marschall, M., Rühl, E., Kästner, B. & Elster, C. Compressive nano-FTIR chemical mapping. *Meas. Sci. Technol.* **33**, 035402. 10.1088/1361-6501/ac407a (2021). 10.1088/1361-6501/ac407a

[32] Tošić, I. & Frossard, P. Dictionary learning. *IEEE Signal Process. Mag.* **28**, 27–38. 10.1109/MSP.2010.939537 (2011). 10.1109/MSP.2010.939537

[33] Rubinstein, R., Bruckstein, A. M. & Elad, M. Dictionaries for sparse representation modeling. *Proc. IEEE* **98**, 1045–1057. 10.1109/JPROC.2010.2040551 (2010). 10.1109/JPROC.2010.2040551

[34] He, H. *et al.* Collaborative low-rank matrix approximation-assisted fast hyperspectral Raman imaging and tip-enhanced Raman spectroscopic imaging. *Anal. Chem.* **93**, 14609–14617. 10.1021/acs.analchem.1c02071 (2021). 34694779 10.1021/acs.analchem.1c02071

[35] Mohseni-Sehdeh, S. & Babaie-Zadeh, M. A fast dictionary-learning-based classification scheme using undercomplete dictionaries. *Signal Process.*[SPACE]10.1016/j.sigpro.2023.109124 (2023). 10.1016/j.sigpro.2023.109124

[36] Laitrakun, S. *et al.* Toward practical augmentation of raman spectra for deep learning classification of contamination in hdd. *J. Inf. Commun. Converg. Eng.* **21**, 208–215 (2023). 10.56977/jicce.2023.21.3.208

[37] Pati, Y. C., Rezaiifar, R. & Krishnaprasad, P. S. Orthogonal matching pursuit: Recursive function approximation with applications to wavelet decomposition. In *Proc. 27th Asilomar conference on signals, systems and computers*, 40–44, 10.1109/ACSSC.1993.342465 (1993).

[38] Xu, B., Wang, N., Chen, T. & Li, M. Empirical evaluation of rectified activations in convolutional network. arXiv: 1505.00853 (2015).

[39] Ma, K., Van de Voort, F., Ismail, A., Zhuo, H. & Cheng, B. Monitoring peroxide value in fatliquor manufacture by Fourier transform infrared spectroscopy. *J. Am. Oil Chem. Soc.* **77**, 681–685. 10.1007/s11746-000-0109-2 (2000). 10.1007/s11746-000-0109-2

[40] Cabernard, L., Roscher, L., Lorenz, C., Gerdts, G. & Primpke, S. Comparison of Raman and Fourier transform infrared spectroscopy for the quantification of microplastics in the aquatic environment. *Environ. Sci. Technol.* **52**, 13279–13288. 10.1021/acs.est.8b03438 (2018). 30350953 10.1021/acs.est.8b03438

[41] Nim, B., Opaprakasit, M., Petchsuk, A. & Opaprakasit, P. Microwave-assisted chemical recycling of polylactide (PLA) by alcoholysis with various diols. *Polym. Degrad. Stab.* **181**, 109363. 10.1016/j.polymdegradstab.2020.109363 (2020). 10.1016/j.polymdegradstab.2020.109363

[42] Zhang, X. *et al.* A new approach to removing interference of moisture from FTIR spectrum. *Spectrochim. Acta A Mol. Biomol. Spectrosc.* **265**, 120373. 10.1016/j.saa.2021.120373 (2022). 34547685 10.1016/j.saa.2021.120373

[43] Dai, Y. *et al.* Hybrid principal component analysis denoising enables rapid, label-free morpho-chemical quantification of individual nanoliposomes. *Anal. Chem.* **94**, 14232–14241. 10.1021/acs.analchem.2c02518 (2022). 36202399 10.1021/acs.analchem.2c02518

[44] Chen, J., Lu, M., Chen, X., Chen, J. & Chen, L. A spectral gradient difference based approach for land cover change detection. *ISPRS J. Photogramm. Remote. Sens.* **85**, 1–12. 10.1016/j.isprsjprs.2013.07.009 (2013). 10.1016/j.isprsjprs.2013.07.009

